# Laparoscopic ventral hernia repair: A surgeon's insights into defect closure

**DOI:** 10.1016/j.sipas.2025.100280

**Published:** 2025-03-19

**Authors:** Muayad Abass Fadhel

**Affiliations:** Surgery Department, College of Medicine, University of Baghdad, Bab Almoadhem, Medical City Complex 61035/ 00964, Baghdad, Iraq

**Keywords:** Ventral hernia, Laparoscopic ventral hernia repair (LVHR), Defect closure, Mesh

## Abstract

**Introduction:**

Laparoscopic ventral hernia repair (LVHR) is favored over open-repair methods due to reduced pain, shorter hospital stays, and lower recurrence rates. However, whether to close the hernial defect before placing the mesh remains debated. This study compares LVHR outcomes with and without defect closure.

**Methods:**

A retrospective analysis was conducted on 108 patients undergoing LVHR from 2018 to 2022. Group A (49 patients) underwent LVHR without defect closure, and Group B (59 patients) underwent LVHR with defect closure. Data on demographics, operative time, complication rates, postoperative pain, and hernia recurrence were analyzed. Patients were followed for at least 1 year after surgery.

**Results:**

Closing the hernial defect significantly reduced seroma formation (P = 0.04) and mesh bulge (P = 0.04). However, it was associated with longer operative times and higher immediate postoperative pain. Group A had shorter operative times and lower initial pain but higher rates of seroma formation (18.4 %) and mesh bulge (6.1 %). Group B had fewer seromas (5.1 %) and no mesh bulges but required longer surgeries and reported higher immediate postoperative pain. No significant differences were observed in hernia recurrence rates, hospital stays, or chronic pain levels. Mesh infections were only noted in the non-closure group (2.0 %).

**Conclusions:**

Defect closure during LVHR reduces seroma formation and mesh bulge but increases operative time and immediate postoperative pain. Further randomized controlled trials are necessary to optimize LVHR protocols.

## Highlights

• Defect Closure Benefits: Reduces seroma formation and mesh bulge.
• Defect closure issues: Prolong surgery time, increased immediate postoperative pain.
• Outcomes: No difference in hernia recurrence, hospital stay, or chronic pain rates.
• Infection Risk: Mesh infections only observed in the non-closure group.

## 1 Introduction

Ventral hernias occur in anywhere from 3 to 29 % of patients seen by surgeons [1]. Techniques to repair hernias date back to 1993 [1]. The most common method entails positioning the mesh over the defect without closure [2]. Laparoscopic ventral hernia repair (LVHR) has become an increasingly popular technique for repairing hernias [3]. It has advantages such as decreased pain, shorter hospital stays, and lower chances of recurrence compared with open-repair methods [4]; however, LVHR is not standardized.

One of the most debated aspects of this technique is whether to close the defect (i.e., the hole or weak spot in the muscle layer) before placing the mesh [5]; this decision has several significant implications. Closing the defect (also known as primary closure) involves suturing the edges of the defect before mesh placement. This technique is believed to reduce the formation of seroma (i.e., the accumulation of fluid in the space created by the hernia) and prevent 'mesh bulge,' which can lead to patient discomfort and dissatisfaction, reduced recurrence rate, improved abdominal wall function, and enhanced abdominal wall contour postoperatively [6]. However, primary closure is technically more challenging and may increase the operative time and risk of complications, such as wound infection and suture pull-through [7].

However, not closing the defect simplifies the procedure and may reduce the operative time [8]. The mesh bridges the defect and provides tension-free repair, a fundamental principle in hernia surgery for preventing recurrence [8]. Not closing the defect is associated with higher rates of seroma and mesh bulge [8].

This study hypothesizes that compared with non-closure, closure of the hernia defect during LVHR reduces postoperative complications — such as seroma formation and mesh bulge — without increasing hernia recurrence.

Herein, I provide novel data to inform this ongoing debate. By sharing this single-center experience and analyzing the outcomes of LVHR conducted in Iraq, I provide evidence-based recommendations to optimize patient outcomes in ventral hernia repair by focusing on critical outcomes, such as hernia recurrence, complication rates, operative time, hospital stay duration, postoperative pain, and chronic pain affecting quality of life.

## 2 Material and methods

The retrospective comparative analysis of LVHR was conducted at Baghdad Teaching Hospital. Hernia cases treated between January 2018 and December 2022 were considered, with each patient followed up for at least 1 year. Patients aged >18 years with a hernial defect measuring <8 cm were included in the study. Exclusion criteria involved patients with a hernial defect >8 cm, those unable to undergo general anesthesia, individuals with recurrent ventral hernias following laparoscopic surgery, hernias with defects <1 cm, and those lost to follow-up. A total of 108 eligible individuals underwent LVHR with composite on-lay mesh during the study period. The patients were divided into two groups: the first group included 49 patients who underwent laparoscopic placement of an intraperitoneal mesh for hernia defect repair without prior closure of the defect; the second group consisted of 59 patients who underwent laparoscopic placement of an intraperitoneal mesh for hernia defect repair with closure of the defect prior to mesh application. Patients were divided into groups based on evolving procedures at the institution. Initially, individuals were enrolled in the first group, representing the previous practice of not closing the hernial defect during mesh application. However, as the study progressed, my surgical method changed, and the second group included patients who were treated in the final 2 years of the study period; the second group of patients reflects the updated practice of closing a hernia defect before applying a mesh.

The primary outcome of this study was a reduction in postoperative complications, with a specific focus on minimizing seroma formation and mesh bulge. Secondary outcomes included evaluating operative time, assessing postoperative pain levels using the Numeric Rating Scale (NRS), determining hernia recurrence rates during follow-up, and analyzing the length of hospital stay. Additionally, this study aims to examine the incidence of other complications — such as infections, chronic pain, and deep vein thrombosis (DVT) — to provide a comprehensive evaluation of surgical outcomes.

Data for each patient were tabulated in an Excel sheet (Microsoft, Inc., Redmond, WA, USA), including age, sex, body mass index (BMI), American Society of Anesthesiologists (ASA) classification, previous hernia repairs, the size and location of fascial defects, and comorbidities. In addition, data were collected on operation duration, the size and type of prosthetic mesh implanted, operative complications, length of hospital stay, postoperative complications, conversion rate, and hernia recurrence on extended follow-up. The data were gathered following clinical examination, with consent obtained from all patients. Routine measurements — including blood tests, abdominal ultrasound, and if necessary abdominal computerized tomography (CT) — were performed.

### 2.1 Surgical techniques

#### 2.1.1 Repair without closure

Each patient was placed in a supine position on a table, and their abdominal area was sterilized and partially covered. Carbon dioxide was carefully pumped into the abdomen using a Veress needle whenever feasible or using the Hasson technique. Surgical marks were made. The positions of the trocars differed based on the size and location of the hernia defects. Typically, two 5 mm trocars and one 10 mm trocar were inserted along the side of the abdomen. The bowel and omentum were carefully separated from the wall of the abdomen using a ligasure, and the contents of the hernia were repositioned. The peritoneal sac surrounding the contents was left as is. When the dissection was finished, the size of the hernia gap was measured. In cases of hernias requiring a mesh, a mesh was selected to cover all the edges of the gap by at least 4 cm. Size 0 proline stay stitches were then placed at each corner of the mesh and individually pulled through using a suture passer to ensure the initial fixation of the mesh to the muscle layer. The mesh was put in place using a 10 mm trocar, and a central stitch was used to mark the undersurface of the mesh. Typically, the net was securely attached to the undersurface of the abdominal wall at 1 cm intervals using a hernia tacker. A second row of tacks was added, and other tacks applied as necessary. Next, the trocars were removed, and the patient was observed for bleeding from the trocar sites. The skin incisions were then closed in a subcuticular method using non-absorbable stitches.

#### 2.1.2 Repair with closure

This closure method is similar to that discussed above, but with a slight variation. The defect is closed internally using non-absorbable, barbed, size 0 or 1 sutures, based on the defect size. The suturing process involved using a needle and standard laparoscopic needle grasper to create two rows of sutures. After the defect was closed, an appropriately sized mesh was tailored to overlap all the defects by 4 cm.

Postoperatively, the patients were assessed and discharged. Postoperative pain was evaluated using a 10-point NRS [9]. Pain levels were classified into four categories: none (≤ 1), mild [[2], [3], [4], [5]], moderate [6,7], and severe [[8], [9], [10]].

Patients were postoperatively followed up at 1 week, 3 months, and 1 year. Data on postoperative complications (e.g., seromas, mesh bulge, infections) were collected at each visit. The NRS pain scores were recorded at 24 h, 1 week, and 3 months postoperatively. Twenty patients who were lost to follow-up were excluded from the study. The follow-up was performed by reviewing the data (i.e., the medical records of clinic visits) and conducting structured phone calls.

### 2.2 Data analysis

Data were analyzed using Statistical Package for the Social Sciences (SPSS version 27; IBM Corp., Armonk, NY, USA). Quantitative continuous variables were evaluated by calculating means and standard deviations. Frequencies and percentages were reported for categorical nominal and ordinal variables. Independent t-tests were used to compare continuous variables over time (2010–2015 vs. 2016–2021), while chi-squared tests were employed to assess the association between categorical variables. Statistical significance was defined as P < 0.05.

### 2.3 Ethical considerations

This study adhered to the guidelines of the Declaration of Helsinki and received ethical approval from the Baghdad Teaching Hospital (Approval Number: 422). Written informed consent was obtained from all participants.

## 3 Results

Of the 108 participants, 44 were males and 64 were females. Group A (the non-closure group) comprised 19 males and 30 females; Group B (the closure group) comprised 25 males and 34 females. The average age of the patients in both groups was 40.06 years. Patients in Group A had a mean age of 39.80 years; patients in Group B had a mean age of 42.00 years (Table 1).

**Table 1:** Patient characteristics.

Characteristic	Total	Group A (non-closure)	Group B (defect closure)
n	108	49	59
Male/female	44/64	19/30	25/34
Age ± SD (range) [years]	40.06 ± 7.09 (21–67)	39.80 ± 7.90 (21–65)	42.00 ± 9.12 (25–67)
Mean BMI (range) [kg/m^2^]	30.5 (20.0–55.9)	31.3 (20.0–45.0)	32.9 (28.0–55.0)
Mean ASA score (range)	2.2 (1–4)	2.9 (1–4)	2.3 (1–4)
Previous hernia repair, n (%)	14 (13.0)	9 (18.6)	5 (8.5)
Diseases, n (%)			
DM	27 (25.0)	11 (22.4)	16 (27.1)
HT	36 (33.0)	15 (30.6)	21 (35.6)
IHD	6 (5.6)	2 (3.4)	4 (6.8)
Pulmonary diseases	9 (8.3)	4 (8.2)	5 (8.5)
Thyroid disease	7 (6.5)	4 (8.2)	3 (5.1)

BMI, Body mass index; ASA, American Society of Anesthesiologists; DM, Diabetes mellites; HT, Hypertension; IHD, Ischemic heart disease.

The average BMI of the patients in the cohort was 30.5 kg/m^2^ (range: 20–55 kg/m^2^). Patients in Group A had a mean BMI of 31.3 kg/m^2^ (range: 20–45 kg/m^2^), and patients in Group B had a mean BMI of 32.9 kg/m^2^ (range: 28–55 kg/m^2^). The ASA has proposed a scoring system to gauge the health status of patients, ranging from 1 to 4. The average score of the patients was 2.2; patients in Group A had a higher mean ASA score (2.9) than patients in Group B (2.3). Additionally, 14 patients (13.0 %) had undergone hernia repair surgery in the past: nine patients (18.6 %) in Group A and five patients (8.5 %) in Group B. Common medical conditions among the patients included diabetes mellitus (25.0 %), hypertension (33.0 %), ischemic heart disease (5.6 %), pulmonary diseases (8.3 %), and thyroid diseases (6.5 %). Patients in Group A exhibited a higher prevalence of these medical conditions. These findings provide important insights into the characteristics and medical histories of patients undergoing hernia repair surgery.

**Fig. 1:**
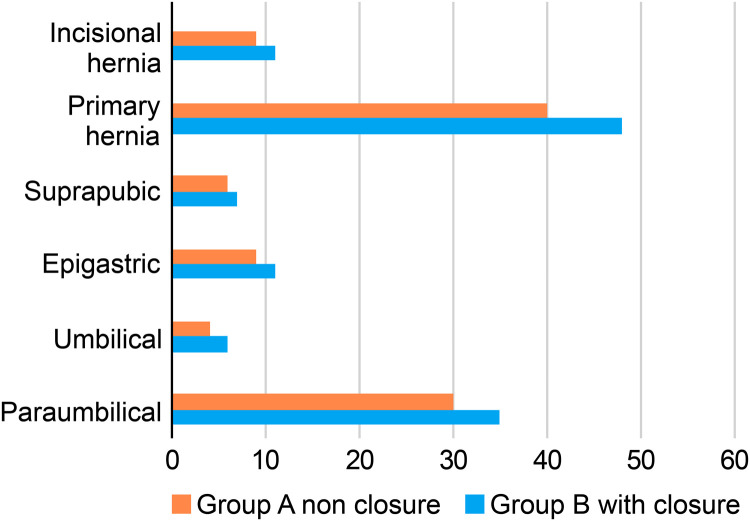
Distribution of the sites and types of hernias in the two patient groups.

Table 2 lists the characteristics of the patients’ hernias and the surgical outcomes of the patient group focusing on hernia location, type, mesh size, operative time, concurrent procedures, hospital stay duration, and conversion rates. Most cases involved paraumbilical hernias (60.2 %), followed by epigastric hernias (18.5 %). Suprapubic hernias accounted for 12.0 % of cases, and umbilical hernias accounted for 9.3 % of cases. The spatial distribution of hernias was similar in both groups and not statistically different (P = 0.06). The types of hernias included primary ventral and incisional sections and were also not statistically different between the two groups (P = 0.06; Fig. 1).

**Table 2:** Hernia characteristics.

Characteristic	N	Group A	Group B	P-Value
Hernia Location				0.06
Paraumbilical, n (%)	65 (60.2)	30 (61.2)	35 (59.3)	
Umbilical, n (%)	10 (9.3)	4 (8.2)	6 (10.2)	
Epigastric, n (%)	20 (18.5)	9 (18.4)	11 (18.6)	
Suprapubic, n (%)	13 (12.0)	6 (12.2)	7 (11.9)	
Hernia type				0.06
Primary ventral, n (%)	88 (81.5)	40 (81.6)	48 (81.4)	
Incisional, n (%)	20 (18.5)	9 (18.4)	11 (18.6)	
Hernia size (cm) n (%)				0.06
2–4	25 (23.2)	14 (28.6)	11 (18.6)	
4–6	62 (57.4)	29 (59.2)	33 (55.9)	
6–8	21 (19.4)	6 (12.2)	15 (25.4)	
Mesh size (cm^2^) mean (range)	186.4 (64–573)	180.2 (70–573)	190.7 (64–500)	0.04
Operative time (min) mean (range)	58.5 (18–143)	45.5 (18–120)	61.0 (35–143)	0.04
Concurrent procedures, n (%)	11 (10.2)	5 (10.2)	6 (10.2)	
Hospital stay (days) mean (range)	2 (1–3)	1 (1–2)	2 (1–3)	0.06
Conversions to open surgery, n (%)	1 (0.9)	0 (0.0)	1 (1.7)	0.06

Hernia sizes can be categorized as 2–4, 4–6, and 6–8 cm. Roughly half of the hernias considered (57.4 %) fell within the range of 4–6 cm, and patients in Group A were more likely to exhibit larger hernias (6–8 cm). The average mesh size was 186.42 cm², and the average operative time was 58.5 min. Patients in Group A experienced shorter procedures than patients in Group B, which may be attributed to the additional time required for the closure procedure used among patients in Group B. Additionally, a difference in mesh size was noted between the two groups: patients in Group B received slightly larger meshes on average (190.8 vs. 180.2 cm²).

Hospital stays ranged from 1 to 3 days; in both groups, the average stay was 2 days. One patient in Group B required conversion to open surgery.

Table 3 lists the complications observed; the most common complication was the development of seromas, which affected 11.1 % of patients. Patients in Group A exhibited a higher rate of occurrence of seromas (18.4 %) than those in Group B (5.1 %), and this difference was statistically significant (P = 0.04). Other complications included wound infections (4.6 %), sepsis (1.0 %), urinary retention (6.5 %), and respiratory issues (4.6 %); however, there were no differences in the prevalence of these complications between the two groups (P = 0.06). Mesh infections were only observed in patients in Group A (2.0 %), and DVT occurred in one patient in each group.

**Table 3:** Postoperative complications.

Complication	N	Group A	Group B	P-Value
Seroma n (%)	12 (11.1)	9 (18.4)	3 (5.1)	0.04
Wound infection n (%)	5 (4.6)	2 (4.1)	3 (5.1)	0.06
Mesh infection n (%)	1 (0.9)	1 (2.0)	0 (0.0)	0.06
Sepsis n (%)	1 (0.9)	1 (2.0)	0 (0.0)	0.06
Urine retention n (%)	7 (6.5)	2 (4.1)	5 (8.5)	0.06
DVT n (%)	1 (0.9)	1 (2.0)	0 (0.0)	0.06
Respiratory complications n (%)	5 (4.6)	2 (4.1)	3 (5.1)	0.06
Ileus n (%)	2 (1.9)	1 (2.0)	1 (1.7)	0.06
Bowel injury n (%)	0	0 (0.0)	0 (0.0)	0.06
Hernia recurrence n (%)	4 (3.7)	3 (6.1)	1 (1.7)	0.06
Chronic pain n (%)	1 (0.9)	0 (0.0)	1 (1.7)	0.06
Mesh bulge n (%)	3 (2.8)	3 (6.1)	0 (0.0)	0.04

DVT, Deep vein thrombosis.

Hernia recurrence was observed in 3.7 % of patients; the intergroup difference was not statistically significant (P = 0.06). Chronic pain was reported by 0.9 % of patients, and the difference between the groups was not statistically different (P = 0.06). However, there was a statistically significant difference in the occurrence of mesh bulge between Groups A and B (P = 0.04); patients in Group A exhibited a higher rate of this complication (6.1 %) compared with those in Group B (0.0 %). I observed significant variations in the development of seroma and mesh bulge between patients who did and did not undergo closure. These results highlight the role of surgical techniques and postoperative care in reducing complications associated with hernia repair.

Table 4 displays the postoperative pain scores over three periods: 24 h, 1 week, and 3 months for Groups A and B. The table shows that at 24 h, 67.3 % of patients in Group A and 64.4 % in Group B reported mild pain, 18.4 % of Group A and 27.1 % of Group B reported moderate pain, and 4.1 % in Group A and 5.1 % in Group B reported severe pain. A significant difference was found between groups in the "None (≤1)" and "Moderate (6–7)" categories (P = 0.04), with Group A having more patients with no pain (10.2 %) and fewer with moderate pain than Group B. By 1 week, most patients reported no pain—95.9 % in Group A and 91.5 % in Group B. A small percentage in Group A (4.1 %) and Group B (8.5 %) reported mild pain, with a significant difference between the groups (P = 0.04).

**Table 4:** Postoperative NRS pain score.

Time Interval	NRS Pain Score	N (%)	Group A, n (%)	Group B, n (%)	P-Value
24 H	None (≤ 1)	7 (6.5)	5 (10.2)	2 (3.4)	0.04
	Mild (2–5)	71 (65.7)	33 (67.3)	38 (64.4)	0.06
	Moderate (6–7)	25 (23.1)	9 (18.4)	16 (27.1)	0.04
	Severe (8–10)	5 (4.6)	2 (4.1)	3 (5.1)	0.06
1 Week	None (≤ 1)	101 (93.5)	47 (95.9)	54 (91.5)	0.04
	Mild (2–5)	7 (6.5)	2 (4.1)	5 (8.5)	0.06
	Moderate (6–7)	0 (0.0)	0 (0.0)	0 (0.0)	–
	Severe (8–10)	0 (0.0)	0 (0.0)	0 (0.0)	–
3 Months	None (≤ 1)	107 (99.1)	49 (100.0)	58 (98.3)	0.34
	Mild (2–5)	1 (0.9)	0 (0.0)	1 (1.7)	0.34
	Moderate (6–7)	0 (0.0)	0 (0.0)	0 (0.0)	–
	Severe (8–10)	0 (0.0)	0 (0.0)	0 (0.0)	–

NRS, Numeric Rating Scale.

Finally, at 3 months, 100 % of patients in Group A and 98.3 % in Group B were pain-free, with one patient experiencing mild pain in Group B; not significant difference was observed between the groups (P = 0.34).

In general, most of the patients analyzed experienced pain; however, there were notable differences in pain severity between the closure and non-closure groups. These results highlight the need for pain-management approaches to enhance patient comfort and recovery after hernia repair surgeries.

## 4 Discussion

There is ongoing discussion regarding whether to close the opening during LVHR. My research focuses on analyzing the outcomes of LVHR at our hospital, thereby contributing to this dialogue.

Patient characteristics — such as age, sex, BMI, and existing health conditions — were examined to assess their influence on outcomes. This approach is consistent with studies that have also explored how patient factors can impact treatment response and long-term health outcomes [9]. While my research did not find differences in LVHR outcomes on the basis of patient characteristics, it would be beneficial for future studies to explore how specific patient factors influence treatment outcomes.

The duration of surgery showed no statistically significant variation based on the LVHR approach used. This finding corresponds with the results of Saijo et al. [10], who noted that there were no significant differences in surgical procedure duration between the two LVHR approaches. However, Husslein et al. [11] noted a significant disparity in surgical duration between patient groups that underwent closure and those that did not. A higher incidence of seroma formation was observed in the non-closure group, consistent with previous research [12]. Shao et al. [12] showed that keeping a defect open could potentially increase the probability of buildup in the sac and elevate the risk of seroma development. Similarly, Bittner et al. [13] noted that closing the defect can decrease the likelihood of seroma formation by enhancing tissue approximation and minimizing dead space.

Mesh infections were only noted in the non-closure group. This finding is consistent with previous research reporting that leaving a defect open may increase the risk of infectious complications. Lanier et al.]^14^] noted that primary closure of a defect can reduce the risk of mesh infections by minimizing contact between the mesh and the contents of the defect; no statistically significant differences in hernia recurrence rates were found between the closure and non-closure groups. This finding is consistent with earlier studies that reported comparable recurrence rates between the two techniques. However, Baker et al. [15] proposed that defect closure may result in lower recurrence rates by enhancing mesh fixation and minimizing tension during repair.

Patients in the closure group reported higher levels of postoperative pain than patients in the non-closure group. This finding is consistent with previous studies that also reported higher pain levels with closure techniques [16]. However, Beldi et al. [17] reported conflicting results, noting no significant difference in postoperative pain between closure and non-closure groups. Chronic pain was reported by a small percentage of patients, consistent with previous studies that also reported low rates of chronic pain following LVHR [18]. However, other studies have reported higher rates of chronic pain, which indicates variability in patient experiences and potential contributing factors, such as surgical technique and patient characteristics [19].

Both the closure and non-closure groups in this study exhibited similar durations of hospital stay. This is consistent with previous research that found no significant difference in hospital stay between the two techniques [20]. This finding suggests that both techniques are associated with comparable postoperative recovery times and resource utilization [20]. One patient in the closure group required a switch to open surgery. This finding is consistent with other studies that have demonstrated instances of requiring conversion to open surgery during laparoscopic hernia repair procedures [21]. While the need for surgery is rare, it underscores the importance of having surgeons who are ready to manage unexpected issues [21].

The patient cohort included 20 individuals who were lost to follow-up, highlighting a challenge of observational research. This challenge is not exclusive to this study, and other researchers have faced similar issues [22]. Accordingly, it is important to establish follow-up procedures and define efficient strategies to minimize the probability of losing participants, as well as address biases that may affect the study results [22].

My findings provide insights into the side effects of various LVHR methods. To support evidence-based approaches, it is crucial to conduct additional randomized controlled trials focused on hernia repair to confirm the validity of these results.

## 5 Conclusions

Closing the hernial defect during LVHR reduces complications such as seroma formation and mesh bulge; however, the procedure is technically more challenging and results in higher levels of immediate postoperative pain. No significant differences were observed in hernia recurrence rates, hospital stay durations, or chronic pain levels between the closure and non-closure groups; however, additional randomized controlled trials are necessary to validate these findings. Surgeons conducting LVHR should weigh reduced complication rates against increased operative challenges and implement enhanced pain management for patients undergoing defect closure.

## Data availability

The datasets generated and/or analyzed during the current study are not publicly available due ethical restrictions but are available from the corresponding author on reasonable request.

## Ethics approval

This study adhered to the guidelines of the Declaration of Helsinki and received ethical approval from the Ethics Committees of Baghdad Teaching Hospital (Approval Number: 422).

## Funding

This research did not receive any specific grant from funding agencies in the public, commercial, or not-for-profit sectors.

## CRediT authorship contribution statement

**Muayad Abass Fadhel:** Writing – review & editing, Writing – original draft, Visualization, Validation, Supervision, Software, Resources, Project administration, Methodology, Investigation, Formal analysis, Data curation, Conceptualization.

## Declaration of competing interest

The author declares that he has no known competing financial interests or personal relationships that could have appeared to influence the work reported in this paper.
